# Microscopic study of the intermediate mixed state in intertype superconductors

**DOI:** 10.3762/bjnano.17.5

**Published:** 2026-01-07

**Authors:** Vyacheslav D Neverov, Alexander V Kalashnikov, Andrey V Krasavin, Alexei Vagov

**Affiliations:** 1 Moscow Institute of Physics and Technology, 141700 Dolgoprudny, Russian Federation; 2 HSE University, Moscow 101000, Russian Federation; 3 National Research Nuclear University MEPhI, Moscow 115409, Russian Federation

**Keywords:** Bogoliubov–de Gennes equations, intertype regime, microscopic calculations, superconductivity, vortices in superconductors

## Abstract

We present a comprehensive microscopic study of the intermediate mixed state in superconductors of the intertype (IT) regime separating types I and II. Using fully self-consistent Bogoliubov–de Gennes calculations for a lattice model, we analyze few-vortex configurations across the entire temperature range 0 *< T < T*_c_. Our results demonstrate the key features of IT superconductivity, namely, nonmonotonic vortex interactions and formation of vortex clusters. Using results of the calculations, we construct a “temperature–coupling” phase diagram that delineates distinct superconducting regimes and shows their convergence at a single Bogomolnyi point, consistent with earlier predictions of extended Ginzburg–Landau theory. Additionally, we identify a deep IT region of irregular vortex configurations apparently dominated by many-body vortex effects. The results establish a fully microscopic foundation for the IT superconductivity and extend its description beyond the vicinity of the critical temperature.

## Introduction

The magnetic response of superconductors has long served as a fundamental criterion for their classification into distinct types. Traditionally, two types are recognized, namely, type I, in which magnetic fields are completely expelled from the material (the Meissner state), and type II, with magnetic flux penetration in the form of quantized vortices forming a mixed state [1–3]. Within the Ginzburg–Landau (GL) framework, the boundary between these regimes is determined by the GL parameter κ = λ_L_/ξ_GL_, where λ_L_ is the magnetic London penetration depth and ξ_GL_ is Ginsburg–Landau coherence length, with the critical value 
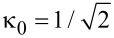
 separating type-I (κ *<* κ_0_) and type-II (κ *>* κ_0_) behavior [4].

However, experimental studies have shown that this traditional dichotomy is incomplete even for superconductors with a single gap function (single-band superconductors). In materials with κ_GL_ close to κ_0_, the magnetic flux penetrates the sample in complex, irregular patterns that cannot be attributed to type-I or type-II behavior [5–16]. These patterns are referred to as intermediate mixed state (IMS) and are characterized by the coexistence of Meissner domains and vortex clusters, chains, or fragmented lattices.

Initially referred to as type II/1 superconductivity [14], this regime has since been understood as a manifestation of a more general intertype (IT) superconductivity [17–20], which fundamentally extends the conventional classification. The physics of IT superconductivity is closely related to the infinite degeneracy of the superconducting state at the so-called Bogomolnyi (ℬ) point (κ_0_, *T*_c_) with *T*_c_ being the critical temperature [21–22]. At this point, the surface energy between the superconducting and normal phases vanishes, allowing for a continuum of flux–condensate configurations with equal energy. Deviations from the ℬ point lift this degeneracy, creating a finite IT domain in the (κ, *T*) phase diagram [17,23–24]. Within this domain, the system supports a variety of states with close energies that feature nonuniform flux distributions and complex vortex arrangements.

Based on the perturbation expansion of the BCS theory, also referred to as the extended Ginzburg-Landau (EGL) formalism [23–25], it has been demonstrated that the emergence of IT behavior is a universal phenomenon and occurs in both single- and multiband superconductors [17]. One of its key features is nonmonotonic vortex–vortex interactions, which are attractive at long and repulsive at short ranges [14]. The long-range attraction destabilizes the regular Abrikosov lattice, promoting the formation of vortex clusters. Subsequent studies have also highlighted the important role of many-vortex effects, which extend beyond simple pairwise interactions and decisively shape IMS vortex configurations [26–27].

Despite the long-standing experimental evidence and theoretical efforts, IT superconductivity remains insufficiently explored. This gap arises from the limitations of perturbative approaches, which are strictly valid only in the vicinity of ℬ close to *T*_c_. Although higher-order expansions of the BCS theory beyond the GL level successfully describe certain features of the IMS, a fully microscopic description applicable across the entire temperature range has been lacking until now. Recent zero-temperature calculations within the Bogoliubov–de Gennes (BdG) framework [1] have demonstrated IT behavior by studying few-vortex configurations, revealing the coexistence of repulsion and attraction that leads to vortex clustering [28].

In this work, we extend these microscopic BdG calculations to the entire temperature range 0 *< T < T*_c_ and investigate the evolution of few-vortex states as the system changes between type-I and type-II regimes. Our results show that the key qualitative features of the IT superconductivity persist throughout this range; however, the IT domain gradually narrows as the temperature increases, shrinking to a single point at *T*_c_. Based on these findings, we construct a phase diagram of the IT regime, which appears qualitatively consistent with that obtained earlier from perturbation theory for the conventional BCS model with a spherical Fermi surface.

## Results and Discussion

### Model and method

The vortex configurations are analyzed within a microscopic lattice model of a superconductor described by the attractive Hubbard Hamiltonian:


[1]

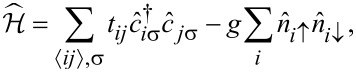



where 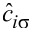 (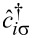) are the annihilation (creation) operator for an electron with spin σ at site *i*, 
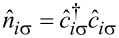
 is the electron number operator, *t_ij_* = *−t* is the nearest-neighbor hopping amplitude, and *g >* 0 denotes the onsite attraction strength. An external magnetic field is incorporated via the Peierls substitution in the hopping matrix elements as


[2]

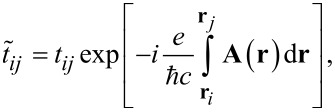



where **A**(**r**) is the vector potential associated with the magnetic field **B** = ∇ × **A**.

Within the mean-field approximation, the superconducting state is determined by solving the BdG equations [1,29]:


[3]

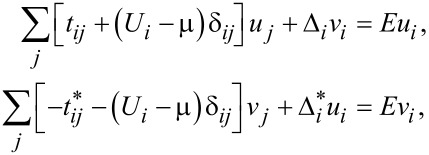



where *u_i_* and *v_i_* are the particle and hole components of the quasiparticle wave function, and μ is the chemical potential. The superconducting gap Δ*_i_* and Hartree–Fock potential *U_i_* are obtained self-consistently from


[4] $$\begin{array}{c} \Delta_{i}=g\sum_{n}u_{i}^{\left(n\right)}v_{i}^{(n)*}\tanh\left(\frac{E_{n}}{k_{\text{B}}T}\right),\\ U_{i}=g\sum_{n}\left[{\left|u_{i}^{\left(n\right)}\right|}^{2}f\left(E_{n}\right)+{\left|v_{i}^{\left(n\right)}\right|}^{2}f\left(-E_{n}\right)\right], \end{array}$$

where *n* labels the BdG eigenstates, and *f*(*E*) is the Fermi–Dirac distribution function. The electron density


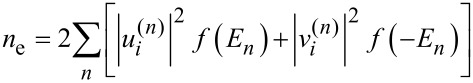


is kept constant at *n*_e_ = 0.25 throughout all calculations by adjusting the chemical potential μ. Notice that the system is well away from the resonance at *n*_e_ = 1 and, at the chosen value, the electronic dispersion is well approximated by a quadratic dependence.

The BdG equations are coupled self-consistently to the magnetic field through the Ampère–Maxwell law, expressed in the Biot–Savart form for the induced vector potential **A**_ind_:


[5] $$A_{\text{ind}}\left(r\right)=\int_{V}\frac{j(r^{\prime },A)}{\left|\left|r-r^{\prime }\right|\right|}{\text{d}}^{3}r^{\prime }.$$

The total vector potential is **A** = **A**_0_ + **A**_ind_, where **A**_0_ corresponds to the uniform external field. The current density **j**, defined on the links between neighboring sites *i* and *j*, is given by


[6]

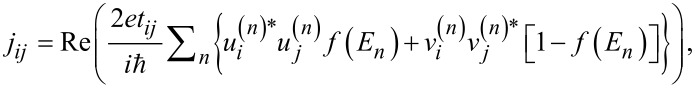



where the Peierls phase in *t_ij_* ensures coupling to the magnetic field. The coupled system of Equation 3–Equation 6 is solved self-consistently using an iterative algorithm developed in [28,30].

The calculations are performed for a 3D slab geometry, finite in the *xy*-plane with size *N* × *N* and infinite along the *z*-axis. The magnetic field **B** = (0,0,B) is applied along the *z*-axis, rendering the problem effectively 2D, except for the Biot–Savart equation (Equation 5), where the integral remains 3D.

In the calculations we set *u,v* = 0 at the boundaries of the system often referred to as “open boundary conditions”. We consider a sample with the relatively small linear size of *N* = 31 due to the high computational cost of achieving convergence with respect to both the superconducting gap and the magnetic field. However, this length exceeds the characteristic superconducting coherence length, which limits the influence of the finite-size effects. The electron density along *z* is absorbed into the parameters of the BdG and Biot–Savart equations. All energies are expressed in units of the hopping amplitude *t*, and lengths are measured in units of the lattice constant *a*.

To identify the superconductivity type, we analyze vortex configurations obtained from self-consistent microscopic calculations. We focus on configurations containing three vortices, which is sufficient to capture both vortex clustering and multivortex (many-body) interaction effects while remaining computationally feasible.

### Vortex configurations

**Figure 1:**
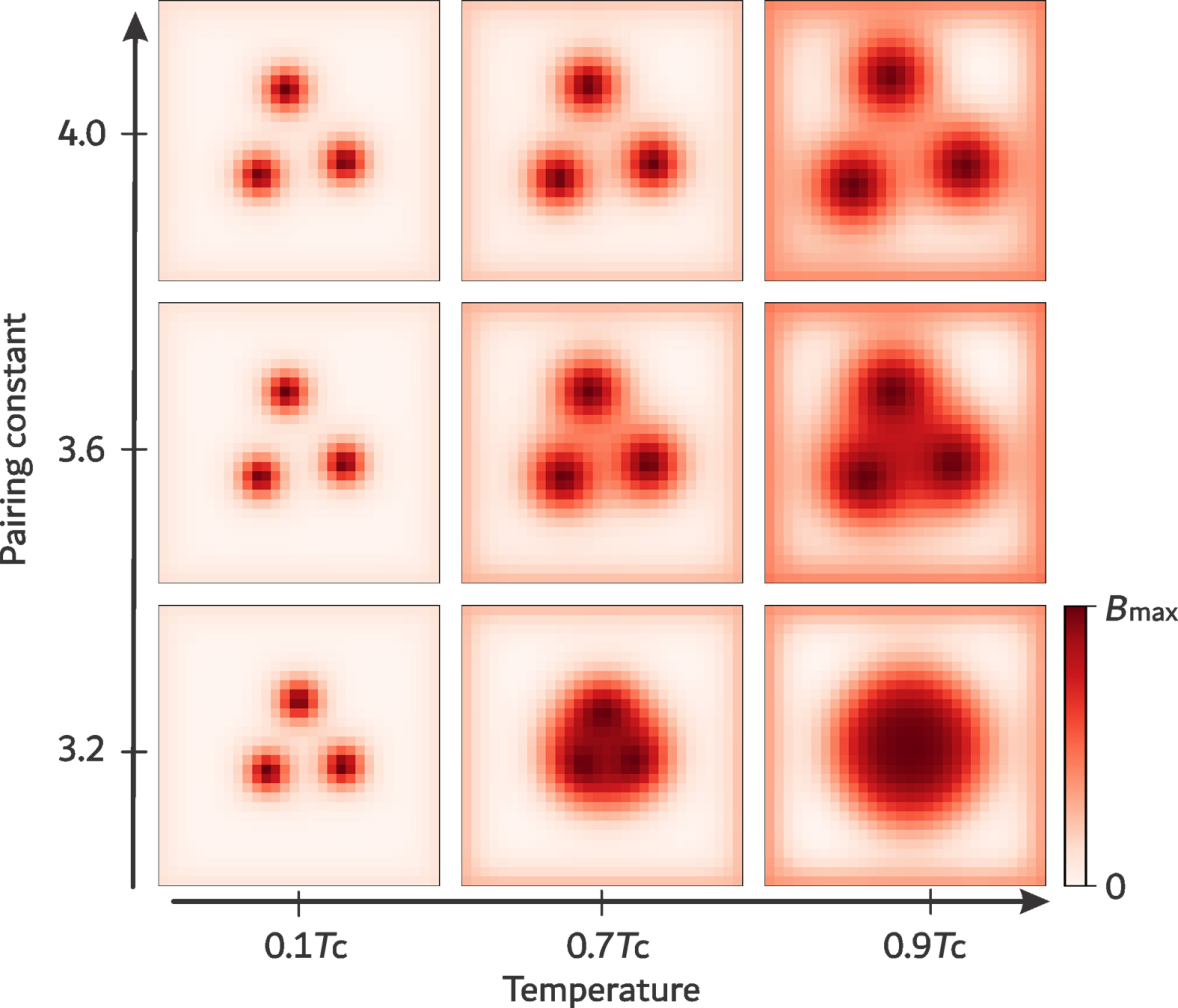
Spatial profile of the magnetic field inside the sample for the IT/2 regime, showing three-vortex configurations computed for pairing constants *g* = 3.2, 3.6, and 4.0 at temperatures *T*/*T*_c_ = 0.1, 0.7, and 0.9, respectively. The external magnetic field is defined as *H* = 3Φ_0_/*N*^2^ where Φ_0_ denotes the superconducting flux quantum.

**Figure 2:**
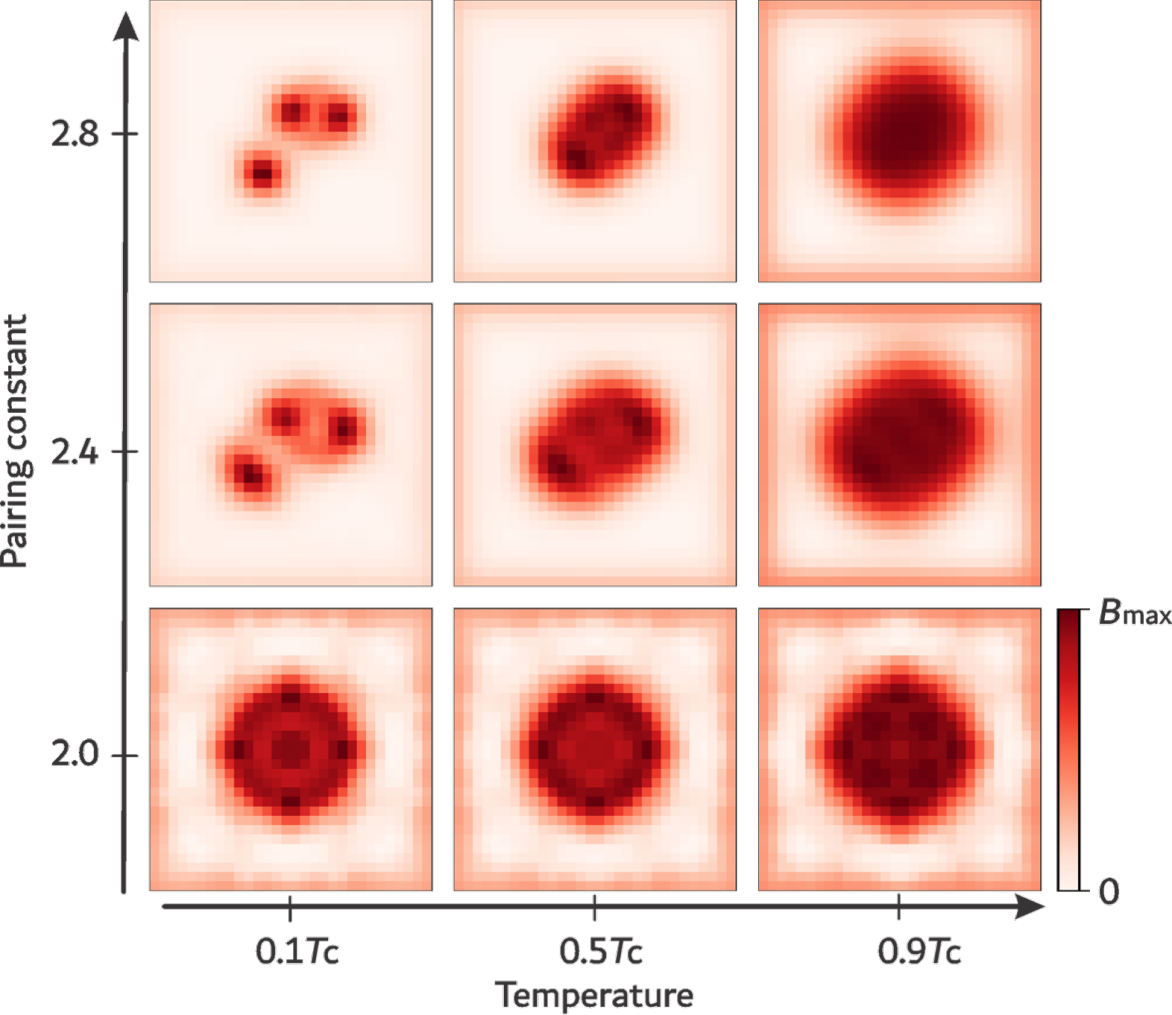
Spatial profile of the magnetic field inside the sample for the IT/1 regime, showing three-vortex configurations computed for pairing constants *g* = 2.0, 2.4, and 2.8 at temperatures *T*/*T*_c_ = 0.1, 0.5, and 0.9, respectively. The external magnetic field is defined as *H* = 3Φ_0_/*N*^2^, where Φ_0_ denotes the superconducting flux quantum.

The results of these calculations are presented in Figure 1 and Figure 2, which display the minimal-energy three-vortex configurations for representative values of the pairing constant *g* and temperature *T*. The variation of *g* and *T* modifies both the coherence length ξ and the magnetic penetration depth λ, and hence their ratio κ = λ/ξ, which determines the superconductivity type. Within the GL theory, ξ and λ share the same temperature dependence, ξ,λ ∝ (1 − *T*/*T*_c_)^−1/2^, making the superconductivity type temperature-independent. In contrast, microscopic theory allows for distinct temperature dependencies of these characteristic lengths, so the type of superconductivity may vary with *T*. This effect is clearly visible in both Figure 1 and Figure 2.

At strong coupling (*g* = 4.0) and high temperature (*T* = 0.9*T*_c_), vortices form an equilateral triangle with maximal inter-vortex separation, which is typical for type-II superconductivity. As the temperature decreases to *T* = 0.1*T*_c_, the triangular arrangement persists, but the inter-vortex distance shrinks, indicating the appearance of a minimum in the vortex–vortex interaction potential. This behavior indicates the coexistence of long-range attraction with short-range repulsion, which is characteristic of the IT superconductivity regime.

At weaker coupling (*g* = 3.2), the system exhibits type-I behavior at high temperature: The three vortices merge into a single giant vortex at *T* = 0.9*T*_c_. As *T* decreases, this giant vortex splits, eventually forming a compact cluster of three vortices at *T* = 0.1*T*_c_, signaling a crossover from type-I to IT superconductivity. Notably, at *T* = 0.1*T*_c_, the vortices again arrange into an equilateral triangle.

The case *g* = 3.6, shown in the middle row of Figure 1, corresponds to the boundary between the two different IT regimes. In this case, vortices form an equilateral triangle, revealing the IT character of the vortex state at all the temperatures considered. However, the inter-vortex separation is smaller than that for the type-II regime (*g* = 4.0, *T* = 0.9*T*_c_), which is consistent with the crossover behavior. Therefore, the high-temperature results (*T* = 0.9*T*_c_) clearly demonstrate how decreasing *g* drives the system from type-II to type-I superconductivity: Isolated Abrikosov vortices gradually merge into a giant vortex also referred to as lamella.

Figure 2 shows results for lower coupling values (*g* = 2.0–2.8), where the system approaches the type-I superconductivity regime. At high temperature (*T* = 0.9*T*_c_), vortices coalesce into a giant vortex for all *g* in this interval. For the weakest coupling (*g* = 2.0), the system remains in the type-I regime at all temperatures. Increasing *g* induces a transition from type-I to IT behavior upon cooling, manifested by the splitting of the giant vortex into single Abrikosov vortices. In this case, however, the vortex configuration becomes asymmetric: Two vortices are closer together than the third. Similar asymmetric arrangements have been previously reported in zero-temperature calculations [30] and have been attributed to enhanced effects of many-body interaction.

### Phase diagram

**Figure 3:**
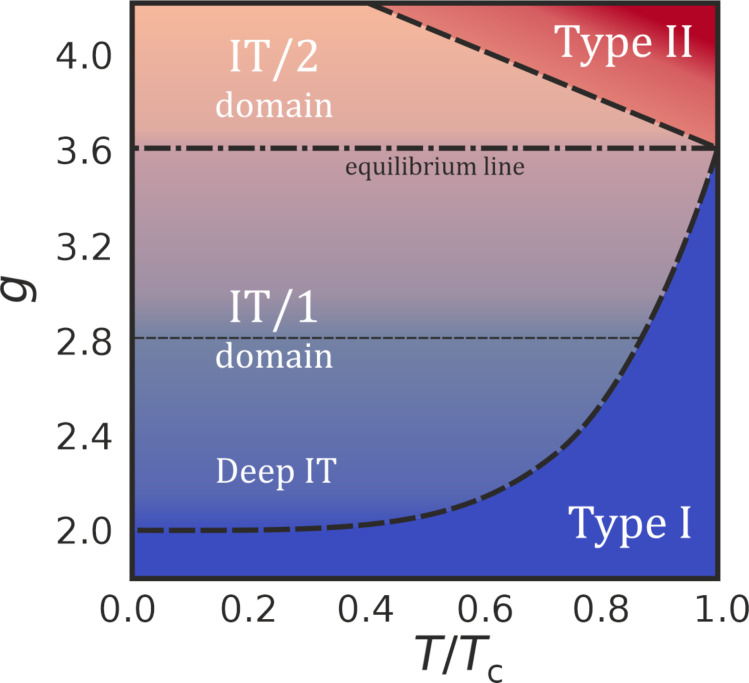
Temperature–coupling (*T*–*g*) phase diagram of vortex matter showing the transition from type I to type II via the IT superconducting regime. The IT type is subdivided into the IT/2 regime with the pairwise vortex interaction and the IT/1 regime with the many-body contribution to the vortex interaction. The thin dashed line separates the “Deep IT” region characterized by significant many-body interaction of vortices.

The complete set of calculations for all values of *g* and *T* is summarized in the phase diagram shown in Figure 3. The diagram reveals three distinct regimes, namely, conventional type-I and type-II superconductivity and the IT regime, where Abrikosov vortices form nonstandard configurations.

The phase diagram shows that the IT regime occupies a larger interval of coupling values *g* at smaller temperatures so that, at *T* = 0, this interval is widest. It narrows as temperature increases, disappearing in the limit *T*→*T*_c_, where all three superconductivity types, type I, type II, and IT, meet at a single point. This critical point has previously been discussed within the EGL expansion of BCS theory and is called the ℬ point, at which the GL parameter takes the value κ = κ_0_. In this work, the ℬ point corresponds to *g* = 3.6, which is indicated as the “equilibrium” line in Figure 3. For *g >* 3.6, the system exhibits a crossover between the type-II and IT regimes, while, for *g <* 3.6, the crossover is between the type-I and IT regimes.

The phase diagram in Figure 3 closely resembles that obtained within the EGL formalism in the κ–*T*-plane, where the GL parameter κ is related to the coupling strength *g*. The sequence of regimes, type I→IT→type II, persists at all temperatures. Thus, microscopic calculations reproduce the known topology of superconductivity-type transitions and also extend it beyond the near-*T*_c_ regime to the entire temperature interval 0 *< T < T*_c_.

We note that, at lower temperatures (
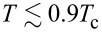
), the microscopic results reveal a subdivision within the IT regime: Around *g* ≃ 2.8, the vortex structure changes from equilateral triangular arrangements (IT/2 domain) to irregular, asymmetric configurations (IT/1 domain) indicating the increasing role of many-body interactions. Similar structural changes were also observed in perturbative analysis, though they become especially apparent in the microscopic study of three-vortex configurations presented here.

## Conclusion

This work presents a fully microscopic analysis of the intermediate mixed state in IT superconductors between the type-I and type-II regimes. Using self-consistent Bogoliubov–de Gennes simulations, we traced the evolution of vortex configurations throughout the temperature range from zero temperature to critical temperature *T*_c_.

Our results demonstrate that the characteristic features of the IT regime, such as nonmonotonic vortex–-vortex interactions and the emergence of vortex clusters, are not limited to the vicinity of *T*_c_ but persist throughout the entire range of possible temperatures. However, the width of the IT interval in terms of the superconducting coupling strength decreases with increasing temperature, eventually collapsing to a single point (Bogomolnyi point), where type-I and type-II superconductivity merge.

The phase diagram constructed from the microscopic calculations provides a unified view of the superconductivity types and their transitions for any superconducting system, conforming and extending earlier predictions of the perturbation theory. In particular, the results confirm the presence of a deep IT region characterized by complex, non-pairwise vortex interactions that lead to irregular vortex patterns. This finding emphasizes the many-body nature of vortex matter in this regime and highlights the limitations of simplified models based on pairwise interaction.

In general, this study establishes a microscopic foundation for the IT regime in single-band superconductors and clarifies its persistence and transformation with temperature. These insights open a path for future investigations of materials tuned near the Bogomolnyi point, where unconventional vortex structures and collective effects may play a decisive role in superconducting behavior and functionality.

## Data Availability

Data generated and analyzed during this study is available from the corresponding author upon reasonable request.
